# Metasurface-Enhanced Infrared Photodetection Using Layered van der Waals MoSe_2_

**DOI:** 10.3390/nano15120913

**Published:** 2025-06-12

**Authors:** Jinchun Li, Zhixiang Xie, Tianxiang Zhao, Hongliang Li, Di Wu, Xuechao Yu

**Affiliations:** 1 Key Laboratory of Materials Physics, Ministry of Education, School of Physics, Zhengzhou University, Zhengzhou 450001, China; jcli2023@sinano.ac.cn; 2 Key Laboratory of Multifunctional Nanomaterials and Smart Systems, Suzhou Institute of Nano-Tech and Nano-Bionics, Chinese Academy of Sciences, Suzhou 215123, China; zxxie2023@sinano.ac.cn (Z.X.); tnx.zhao@gmail.com (T.Z.); hlli2025@sinano.ac.cn (H.L.)

**Keywords:** van der Waals materials, metasurface, surface plasmon resonance, molybdenum diselenide

## Abstract

Transition metal dichalcogenide (TMD) materials have demonstrated promising potential for applications in photodetection due to their tunable bandgaps, high carrier mobility, and strong light absorption capabilities. However, limited by their intrinsic bandgaps, TMDs are unable to efficiently absorb photons with energies below the bandgap, resulting in a significant attenuation of photoresponse in spectral regions beyond the bandgap. This inherently restricts their broadband photodetection performance. By introducing metasurface structures consisting of subwavelength optical elements, localized plasmon resonance effects can be exploited to overcome this absorption limitation, significantly enhancing the light absorption of TMD films. Additionally, the heterogeneous integration process between the metasurface and two-dimensional materials offers low-temperature compatibility advantages, effectively avoiding the limitations imposed by high-temperature doping processes in traditional semiconductor devices. Here, we systematically investigate metasurface-enhanced two-dimensional MoSe_2_ photodetectors, demonstrating broadband responsivity extension into the mid-infrared spectrum via precise control of metasurface structural dimensions. The optimized device possesses a wide spectrum response ranging from 808 nm to 10 μm, and the responsivity (*R*) and specific detection rate (D*) under 4 μm illumination achieve 7.1 mA/W and 1.12 × 10^8^ Jones, respectively. Distinct metasurface configurations exhibit varying impacts on optical absorption characteristics and detection spectral ranges, providing experimental foundations for optimizing high-performance photodetectors. This work establishes a practical pathway for developing broadband optoelectronic devices through nanophotonic structure engineering.

## 1. Introduction

Since the discovery of graphene, van der Waals (vdWs) materials have garnered increasing interest in the scientific community due to their potential applications, with particular attention directed toward transition metal dichalcogenides (TMDs) [1,2]. TMDs have demonstrated significant potential in photodetector applications, especially in terms of their outstanding performance in photoelectric conversion and detection efficiency [3,4,5,6]. In recent years, breakthroughs in material preparation and fabrication techniques have led to continuous improvements in the optoelectronic properties of TMDs, positioning them as an emerging research focus in the field of optoelectronics [7,8,9].

**Figure 1:**
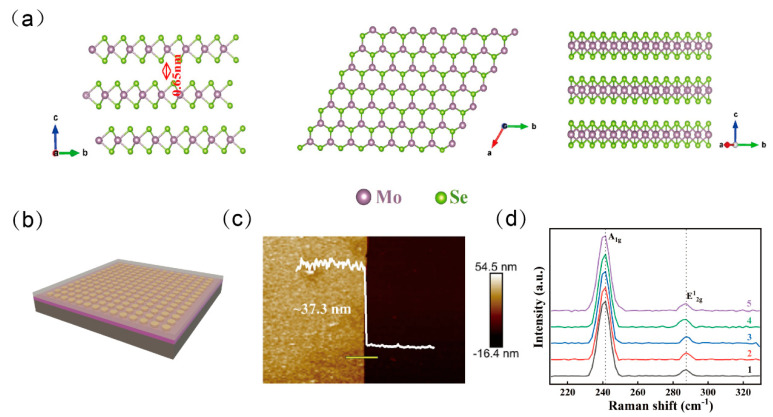
MoSe_2_ and device structure. (**a**) Top and side views of the monoclinic structure of MoSe_2_. (**b**) Schematic diagram of a metasurface-enhanced MoSe_2_ photodetector. (**c**) AFM image of a MoSe_2_ thin film with a measured thickness of approximately 37.3 nm. (**d**) Raman spectra of MoSe_2_ measured at five marked positions in Figure 2a.

Molybdenum diselenide (MoSe_2_), a representative vdWs layered material, possesses notable structural and electronic characteristics [10]. Structurally, monolayer MoSe_2_ exhibits a sandwich-like configuration with a thickness of approximately 0.65 nm, as shown in Figure 1a. Electronically, it displays pronounced quantum confinement effects [11,12]. First principle calculations based on the density functional theory (DFT), incorporating the generalized gradient approximation and spin–orbit coupling (SOC), reveal that monolayer MoSe_2_ possesses a direct bandgap of approximately 1.5 eV. In contrast, this bandgap reduces to around 1.1 eV in the bulk form, indicating a layer-dependent bandgap tunability that opens avenues for band structure engineering [13,14]. Moreover, the absence of dangling bonds on the MoSe_2_ surface provides a unique advantage in constructing heterostructures. When combined with its intrinsic physical and chemical stability, this feature underscores the material’s potential in photodetectors and flexible electronic sensors [15].

However, due to its intrinsic bandgap, the photoresponse of MoSe_2_ films in the mid-infrared (MIR) spectral range is nearly negligible, which severely limits its applications. Eliminating the limitations imposed by the bandgap to enable efficient photoelectric response beyond the intrinsic absorption range of vdWs materials has become a pressing research challenge. An innovative strategy to address this issue lies in leveraging localized surface plasmon resonance (LSPR) effects induced by metasurfaces [16,17,18,19]. By engineering metallic nanostructures, strong localized electromagnetic fields can be generated at specific wavelengths. These fields, through near-field coupling or carrier transfer mechanisms, can extend the photoresponse of materials beyond their inherent absorption spectrum, thereby opening new pathways for the functional enhancement of optoelectronic systems [20,21,22].

In this work, we report a precursor selenization method for the fabrication of large-area, high-quality MoSe_2_ films. Furthermore, we propose the integration of Au-based metasurfaces with MoSe_2_ films to construct a photoconductive Schottky junction detector. By harnessing the LSPR effect of Au nanostructures to generate hot electron injection, this approach effectively overcomes the intrinsic bandgap limitation of MoSe_2_, thereby enabling highly sensitive detection of MIR optical signals. The responsivity and specific detection rate of this device are 7.1 mA/W and 1.12 × 10^8^ Jones at a wavelength of 4 μm. Furthermore, this device has a wide spectral response, ranging from 808 nm to 10 μm. This study not only introduces a novel mechanism for enhancing the photoelectric response of vdWs materials beyond their intrinsic absorption spectrum, but also opens up a new pathway for the design of cost-effective, room-temperature MIR detectors.

**Figure 2:**
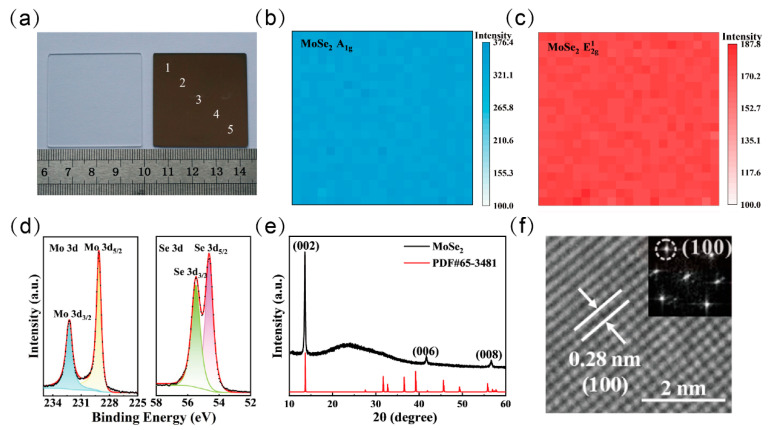
Structural characterization of MoSe_2_ thin films. (**a**) A 4 cm × 4 cm MoSe_2_ film on quartz. (**b**) $A_{1g}$ and (**c**) $E_{2g}^{1}$ Raman mapping images of the MoSe_2_ thin film obtained within an area of 100 μm × 100 μm. (**d**) The XPS spectra and (**e**) XRD patterns of the MoSe_2_ film. (**f**) HRTEM and the corresponding FFT images of MoSe_2_.

## 2. Experiment

### 2.1. Material Synthesis and Characterization

Mo thin films were deposited on SiO_2_ substrates via magnetron sputtering. A gas mixture of hydrogen (5%) and argon was introduced into a tubular furnace at a flow rate of 80 sccm. The furnace temperature was gradually increased to 650 °C and maintained at this temperature for 60 min. During the process, selenium powder was thermally sublimated, generating selenium vapor, which was carried by the Ar/H_2_ gas flow toward the substrate surface. Under high-temperature conditions, the selenium vapor reacted with the Mo thin film, resulting in the formation of a MoSe_2_ film. The synthesized MoSe_2_ films were characterized using a series of techniques, including X-ray diffraction (XRD) (Rigaku, Tokyo, Japan, Smartlab3), Raman spectroscopy (Renishaw, London, UK, InVia), X-ray photoelectron spectroscopy (XPS) (Thermo Scientific, Waltham, MA, USA, ESCALAB Xi^+^), high-resolution TEM (HRTEM) (FEI, Hillsboro, OR, USA, TECNAI G2 F20), and Hall effect measurements to evaluate their structural, compositional, and electrical properties.

### 2.2. Simulation

The optical response of the metasurface structures was simulated using the finite-difference time-domain (FDTD) method. Structural parameters such as disk diameter, period, and thickness were systematically varied to investigate their influence on plasmonic resonance and absorption efficiency. The simulation setup included periodic boundary conditions along the lateral directions and perfectly matched layers (PMLs) in the vertical direction. The refractive index data of MoSe_2_ was measured by means of an ellipsometer, and those for Au and SiO_2_ were obtained from experimentally validated sources to ensure simulation accuracy.

### 2.3. Device Fabrication and Characterization

To fabricate the metasurface-enhanced MoSe_2_ photodetector, PMMA A4 photoresist was first spin-coated onto a SiO_2_ substrate at 4000 rpm for 30 s. Electron-beam lithography was then used to pattern the designed metasurface structure. After exposure, a thin Au/Cr layer was deposited using electron beam evaporation, followed by lift-off to obtain the final plasmonic metasurface. The MoSe_2_ film, synthesized on a separate SiO_2_ substrate, was transferred onto the metasurface using a wet transfer technique. Finally, Au/Cr electrodes were deposited to define a photoconductive channel across the MoSe_2_ film. The electrical and optoelectronic properties of the fabricated device were characterized at room temperature using a measurement system composed of a Keithley 4200-SCS semiconductor parameter analyzer (Tektronix, Beaverton, OR, USA), optical sources, a microscope, and a probe station.

## 3. Results and Discussion

To evaluate the surface morphology, sample quality, and composition of the synthesized MoSe_2_ films, various techniques were employed to analyze the films. As shown in Figure 2a, the large-area MoSe_2_ film fabricated via the selenization method exhibited a uniform color on a 4 cm × 4 cm quartz substrate. Raman spectroscopic analysis (Figure 1d) was performed at five representative points across the sample surface, along with Raman mapping over a 100 μm × 100 μm region provided in Figure 2b,c. The results reveal highly consistent spectral features across all measured regions. Notably, two prominent characteristic peaks located at 240.3 cm^−1^ and 288.1 cm^−1^ correspond to the out-of-plane ($A_{1g}$) and in-plane ($E_{2g}^{1}$) vibrational modes of MoSe_2_, respectively. This high degree of spectral uniformity strongly confirms the excellent homogeneity of the synthesized film.

In Figure 2d, X-ray photoelectron spectroscopy (XPS) analysis of the MoSe_2_ film reveals distinct peaks at binding energies of 228.5 eV and 231.7 eV, corresponding to the Mo 3d_5/2_ and Mo 3d_3/2_ orbitals, respectively. Additionally, a doublet observed at 54.6 eV and 55.4 eV is attributed to the Se 3d_5/2_ and Se 3d_3/2_ orbitals. The presence and relative intensities of these characteristic peaks confirm the stoichiometry of the synthesized film as MoSe_2_. Importantly, no significant impurity-related peaks were detected, indicating a high degree of chemical purity in the sample. As shown in Figure 2e, the X-ray diffraction (XRD) pattern of the film was compared with the standard reference card (PDF#65-3481). Distinct diffraction peaks were observed at 13.7°, 42.1°, and 57.1°, which correspond to the (002), (006), and (008) planes of the MoSe_2_ crystal, respectively. These results confirm that the film possesses a MoSe_2_ phase with high crystallinity and evident preferential orientation, highlighting the structural quality of the synthesized material.

As demonstrated in Figure 2f, high-resolution transmission electron microscopy (HRTEM) imaging coupled with fast Fourier transform (FFT) diffraction analysis confirms that the MoSe_2_ film adopts a monoclinic crystal structure. The observed lattice spacings of 0.28 nm correspond to the (100) crystal planes, respectively. The electrical properties of the MoSe_2_ film were further investigated using a Hall effect measurement system. For a film thickness of 40 nm, the resistivity was measured to be 0.107 Ω·cm, and the carrier mobility reached 8.27 cm^2^·V^−1^·s^−1^. These structural and electrical characterizations collectively confirm the successful synthesis of high-quality 2D MoSe_2_ films.

The metasurface-enhanced MoSe_2_ photodetector, as illustrated in Figure 1b, features a hybrid architecture composed of a MoSe_2_ film integrated with circular plasmonic resonators. The subwavelength design of the metasurface is tailored to match the wavelength of the incident light, enabling the excitation of both surface plasmon polaritons (SPPs) and LSPR. These plasmonic effects result in a strong confinement and concentration of optical energy near the material surface, thereby enhancing the local electromagnetic field. A Schottky junction is formed between the Au metasurface and the MoSe_2_ layer. At resonant wavelengths, the LSPR effect of the Au nanostructures facilitates hot electron injection into the MoSe_2_, effectively overcoming the limitations imposed by its intrinsic bandgap. This mechanism enables enhanced optical absorption and improved photoelectric conversion efficiency, particularly in spectral regions beyond the native absorption range of MoSe_2_.

**Figure 3:**
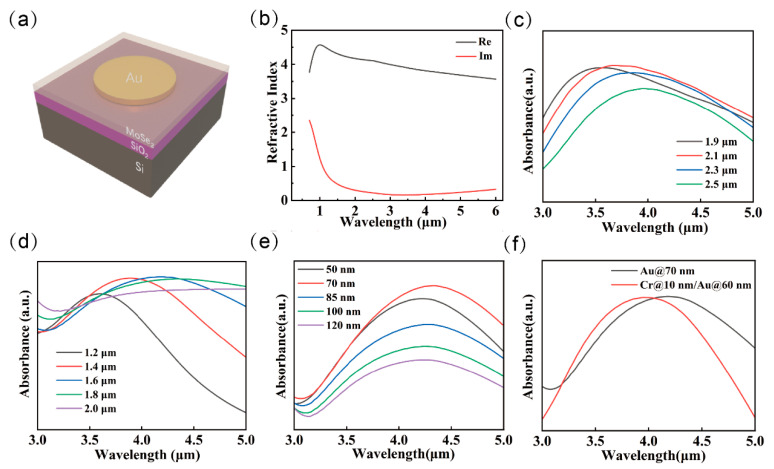
Optical simulation of metasurface structures. (**a**) Schematic diagram of a single-period structure of a metasurface detector. (**b**) Refractive indices of MoSe_2_. (**c**) Passive absorption spectra of the metasurface detectors. The period of the nanodisks was varied while keeping the diameter constant at 1.6 μm. (**d**) Passive absorption spectra of metasurface detectors. The diameter of the nanodisk varies while maintaining a constant period of 2.5 μm. (**e**) Thickness-dependent absorption spectra of the nanodisks. (**f**) Comparison of absorption spectra between pure gold and gold–chromium layer modified gold metasurfaces.

To investigate the optical absorption characteristics of the designed plasmonic structure, finite-difference time-domain (FDTD) simulations were conducted, and the key structural parameters were systematically optimized. Figure 3a presents the schematic of the single-period unit cell configuration set within the simulation software. Based on the approximate resonance condition *SPR* ≈ 2*ne_ff_*, the disk diameter was initially estimated to achieve resonance at the 4 μm wavelength. Subsequently, the effects of metasurface parameters, such as disk diameter and periodicity, on the absorption performance were analyzed through iterative simulations and structural refinements. This optimization process allowed for precise tuning of the plasmonic resonance behavior to maximize optical absorption at the desired spectral position.

When varying the thickness of the metasurface (Figure 3e), distinct absorption behaviors are observed. At a thickness of 50 nm, the metal layer approaches the mean free path of electrons (~40 nm), which enhances surface roughness scattering. Moreover, the film is insufficiently thick to fully confine the electromagnetic field, resulting in increased energy leakage into the substrate or enhanced radiative losses, thereby leading to a relatively low absorption efficiency. At a thickness of 70 nm, the metal layer exceeds three times the skin depth of Au at a 4 μm wavelength (approximately ~20 nm), enabling strong localization of the electromagnetic field. At this point, Ohmic losses have not yet surpassed radiative losses significantly, allowing for the maximization of absorption efficiency. However, further increasing the thickness leads to a dominant contribution from Ohmic losses. An excessively thick metal layer introduces impedance mismatch, which can increase reflection and suppress the efficient excitation of plasmonic resonance modes—such as via cutoff effects in waveguide-like structures—thereby reducing the overall absorption rate.

The observed broadening of the absorption peak with increasing disk diameter, as shown in Figure 3c, primarily arises from the excitation of higher-order multipolar modes and the enhancement of radiative losses. When the disk diameter approaches or exceeds half the effective wavelength (*D* ≥ *λ*/2*n_eff_*), the nano-disk supports not only the fundamental dipolar mode but also higher-order resonances such as quadrupole and magnetic dipole modes. The spectral overlap and superposition of these resonant modes lead to the merging of multiple peaks, resulting in a broadened absorption bandwidth. Moreover, larger structures introduce more radiative decay channels, allowing energy to leak out via enhanced scattering and diffraction. This increased radiative loss reduces the resonance quality factor (Q-factor) and increases the full width at half maximum (FWHM) of the absorption peak. These effects collectively contribute to the broadband absorption characteristics of the plasmonic metasurface.

The redshift of the absorption peak observed with increasing metasurface periodicity is primarily attributed to the change in lattice resonance conditions. For a given period *P*, the resonance wavelength $\lambda_{res}$ approximately satisfies the relationship $\lambda_{res}=P∙n_{eff}$, where $n_{eff}$ is the effective refractive index of the metasurface. At smaller periods (e.g., 1.9 μm), the subwavelength spacing between neighboring unit cells leads to stronger near-field coupling. This enhanced coupling facilitates the excitation of collective resonance modes with stronger energy localization, resulting in higher absorption efficiency. However, as the period increases and the unit spacing exceeds the near-field interaction range (typically on the order of the wavelength), the coupling efficiency declines. Consequently, the collective resonances degrade into localized resonances of individual nanostructures, with increased radiative losses and reduced absorption. Moreover, when the period $P>\lambda /n_{eff}$, higher-order diffraction channels (e.g., m = ±1) become accessible. These channels allow partial energy to escape via diffraction, further lowering the absorption efficiency, as illustrated in Figure 3b.

**Figure 4:**
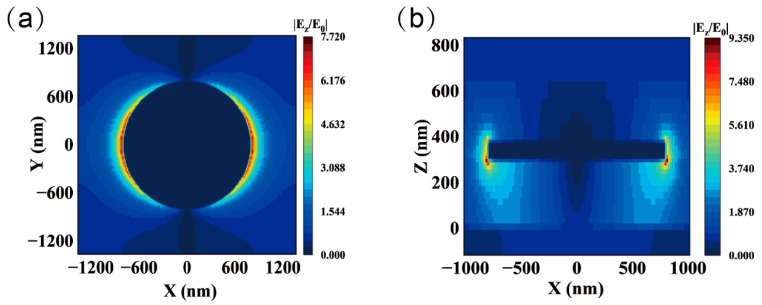
Simulated electric field distributions of the metasurface. (**a**) In-plane electric field distribution. (**b**) Cross-sectional electric field distribution.

For a disk diameter of 1.6 μm, a periodicity of 2.5 μm, and a thickness of 70 nm with Au as the material, the plasmonic resonance peak occurs at a wavelength of 4 μm. The simulated electric field distribution (Figure 4a,b) shows that the field intensity reaches a peak value of approximately $|E/E_{0}|\approx 9.3$, highly localized around the edges of the disks. This ring-shaped high-intensity region indicates the strong confinement of the optical field induced by LSPR. In contrast, the electric field intensity in the gap between adjacent disks is significantly reduced ($|E/E_{0}|<2$), suggesting that the resonance is primarily governed by individual disk elements, with negligible contribution from array coupling effects. Furthermore, the low optical loss of the SiO_2_ substrate suppresses field leakage into the substrate, allowing for greater energy confinement at the disk edges. These observations confirm the metasurface’s precise control over infrared field localization through geometric parameter optimization.

**Figure 5:**
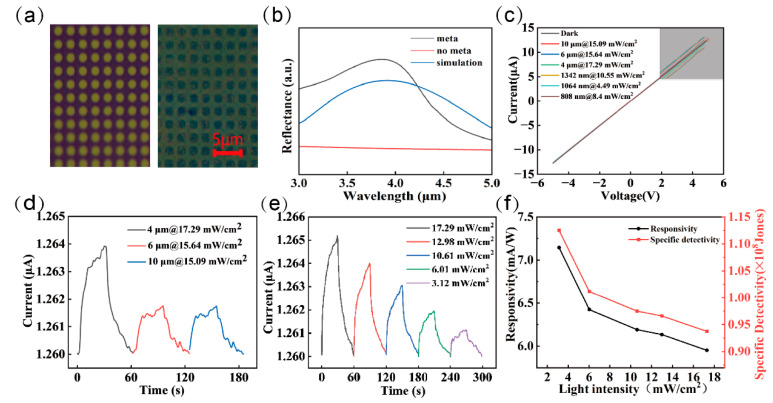
Photoelectric characterization of MoSe_2_ photodetectors enhanced by a metasurface. (**a**) Optical microscopy images of the MoSe_2_ film before and after transfer. (**b**) FTIR reflectance spectra of metasurface detectors. (**c**) *I–V* properties of the photodetector under dark and light irradiation with various wavelengths. (**d**) Time-resolved light response of the device under MIR light irradiation with a bias voltage of 3 V. (**e**) *I–t* curves of the MoSe_2_ device measured under 4 μm light with different intensities at a 3 V bias. (**f**) *R* and D* versus light intensity (4 μm, *V_bias_* = 3 V) in the MoSe_2_ device.

The MoSe_2_ film, synthesized in situ on a SiO_2_ substrate via selenization process, was transferred onto the metasurface array to construct a metasurface-enhanced MoSe_2_ photodetector, as illustrated in Figure 5a,c, which presents the current–voltage (*I–V*) characteristics of the device measured under dark conditions and under illumination at the target wavelength. The *I–V* curves exhibit distinct photoresponse across various infrared wavelength bands, along with a well-defined linear relationship, indicating the formation of an ideal Ohmic contact between the electrodes and the photosensitive MoSe_2_ layer. The formation of an Ohmic contact is critical to the performance of photodetectors. Under ideal Ohmic contact conditions, carrier injection and extraction between the metal electrodes and the semiconductor are not limited by energy barriers, allowing carriers to move freely under an applied electric field. This effectively eliminates the nonlinear rectification behavior typically caused by Schottky barriers. The linearity of the *I–V* characteristics further confirms that the variations in current observed during measurements are purely due to changes in carrier concentration induced by light illumination.

Under illumination at various wavelengths, the device demonstrates significant photoresponse characteristics, with photocurrent enhancement observed across all wavelengths compared to dark conditions. This indicates that photon energy at 808 nm, 1064 nm, 1342 nm, 4 μm, 6 μm, and 10 μm can effectively excite photogenerated carriers, thereby improving the device’s conductivity. The photocurrent–time curves of this metasurface-enhanced photodetector exhibit a typical two-stage rising behavior under all tested wavelengths: an immediate rapid increase upon illumination onset, followed by a slow ascending phase lasting several seconds. This phenomenon suggests the photoresponse process involves multiple physical mechanisms, including transient separation of photogenerated carriers, trap-state release effects, Schottky barrier modulation, and thermal response effects. Additionally, under lower light intensities, the proportion of the slow-rising component in the total photocurrent decreases, indicating its light intensity-dependent contribution.

Upon illumination, photogenerated carriers are promptly injected into the MoSe_2_ and are rapidly separated and transported to the electrodes under the influence of an external bias, leading to a sharp rise in photocurrent. The plasmonic resonance effect of the metallic metasurface further enhances the local electric field, thereby improving the light absorption capability of the vdWs material and significantly increasing the photocarrier generation rate. In addition to optical field enhancement, the metasurface also plays a pivotal role in photocurrent transport through the formation of a Schottky junction between the metal and the MoSe_2_ thin film. Due to the presence of a Schottky barrier, a portion of carriers accumulates near the barrier region, resulting in a relatively high initial photocurrent. Nevertheless, the photocurrent does not reach a steady state immediately after illumination; instead, it enters a slow-rising phase and gradually stabilizes over several seconds. This slow increase is likely related to the release of trap states. A large number of trap states may exist within the MoSe_2_ thin film and at its interfaces, which can capture some of the photogenerated carriers and suppress further photocurrent growth. Under continuous illumination, these trapped carriers are gradually released over time through thermal activation or quantum tunneling, thereby increasing the free carrier concentration and sustaining the continued rise in photocurrent. During the initial illumination phase, a fraction of photogenerated carriers is confined by the Schottky barrier, preventing their immediate participation in conduction. However, as the illumination persists, modulations in the local electric field may lead to a reduction in the effective barrier height, enabling more carriers to overcome the barrier and contribute to conduction, which further enhances the photocurrent.

After the illumination is turned off, the photocurrent does not immediately return to the dark current level, but instead exhibits a noticeable hysteresis effect. This behavior may be primarily governed by the time scale associated with the release of trap states—that is, a portion of the photogenerated carriers remains trapped after the light is removed, and is gradually released over an extended period. Additionally, the slow recovery of the Schottky barrier may influence the decay dynamics of the photocurrent. On the other hand, the relaxation time of the thermal response is relatively long; the gradual decrease in local temperature can lead to a sustained generation of thermally excited carriers, further contributing to the delayed recovery of the photocurrent. These combined effects result in a prolonged decay period following the termination of illumination. To further investigate the photoresponse characteristics of the metasurface-enhanced photodetector under varying power conditions, the device’s I–t response was measured and analyzed under 4 μm wavelength illumination. The experimental results show that the photocurrent increases with increasing optical power, indicating that the device effectively converts optical signals into electrical signals. The experimental results indicate that the device exhibits a distinct photoresponse not only at 4 μm but also at 6 μm and 10 μm, suggesting that the enhancement effect induced by the metasurface is not confined to a single wavelength but extends across a broad spectral range. The simulated electric field distribution in Figure S2 shows that there is also an obvious field enhancement at 6 μm and 10 μm. This phenomenon is likely attributed to the structural parameters of the metasurface, such as its periodicity, duty cycle, and metal thickness. In periodic metasurface structures, the distribution of plasmonic resonance modes can be influenced by the intrinsic properties of the materials, the angle of incidence, and the coupling mechanism of the optical field, thereby resulting in multiple regions of optical enhancement. Moreover, the excitation of higher-order resonance modes may further broaden the spectral range over which enhancement occurs, enabling significant absorption enhancement across a wide portion of the MIR region. This broadband enhancement characteristic greatly improves the practical utility of the photodetector, making it suitable for a wider range of MIR applications.

Under low optical power conditions, the device exhibits a high photoresponsivity and specific detection, reaching up to 7.1 mA/W and 1.12 × 10^8^ Jones. Furthermore, as shown in Figure S3 depicting the device’s 1/f noise power density spectrum, the knee frequency of the device’s *1/f* noise is identified at 966 Hz. Below this knee frequency, the device noise is dominated by a combination of *1/f* noise and *G–R* noise. Above the knee frequency, the noise transitions to being predominantly white noise, which persists across all frequency bands. The device exhibits a noise current density of approximately 10^−21^ A^2^/Hz at high frequencies, where only white noise is present, resulting in low noise levels. Conversely, at low frequencies, the current density reaches 10^−19^ A^2^/Hz, primarily due to the cumulative effect of 1/f noise superposition [23] (Table 1).

However, as the incident power increases, the photoresponsivity gradually decreases. This behavior may be attributed to nonlinear recombination effects of photogenerated carriers at higher power levels [29]. Under low-power illumination, the number of photogenerated carriers is relatively small, and the influence of trap-assisted carrier capture is weak, resulting in higher responsivity. In contrast, under high-power conditions, a larger population of photogenerated carriers may be excited, some of which undergo interband or non-radiative recombination processes, thereby reducing the overall photoresponsivity of the device. Moreover, the plasmonic enhancement effect provided by the metasurface may experience partial saturation or screening at elevated power levels. This could lead to a reduction in the local electric field enhancement, which, in turn, limits the rate of photocurrent increase under high optical power. Such power-dependent behavior underscores the importance of optimizing both the optical and electronic characteristics of the device for efficient operation across a wide range of illumination intensities. Besides excellent performance parameters, atmospheric stability is crucial for device operation. As shown in Figure S4, the MoSe_2_ device maintains its initial photoresponse at 4 μm under 12.98 mW/cm^2^ illumination after two-month ambient storage, demonstrating exceptional operational stability.

## 4. Conclusions

In summary, we successfully synthesized high-quality, thickness-controlled MoSe_2_ films on SiO_2_ substrates via a combination of magnetron sputtering and high-temperature selenization. The metasurface structures were optimized using FDTD simulations, through which we systematically investigated the effects of structural parameters, such as period, thickness, and diameter, on the optical absorption properties of the material. Based on the simulation results, a photoconductive Schottky junction detector was fabricated by integrating an Au metasurface with the MoSe_2_ film. Leveraging the hot carrier injection mechanism induced by the LSPR effect of Au, we successfully overcame the intrinsic bandgap limitation of MoSe_2_ and achieved high-performance photoresponsive detection, reaching up to 7.1 mA/W and 1.12 × 10^8^ Jones at the MIR wavelength of 4 μm. This study not only introduces a novel mechanism for enhancing the photoelectric response of vdWs materials beyond their intrinsic absorption spectrum, but also paves the way for the design of low-cost, room-temperature MIR photodetectors with significant scientific and practical value.

## Figures and Tables

**Table 1:** Performance comparison of MoSe_2_-based photodetectors.

Device	Wavelength	*R* (mA/W)	D* (Jones)	References
MoSe_2_/metasurface	4 μm	7.1	1.12 × 10^8^	This work
MoSe_2_	532 nm	13	/	[24]
MoSe_2_/WSe_2_	520 nm	27.54	/	[25]
MoSe_2_/Si	1100 nm	522	6.7 × 10^9^	[12]
MoSe_2_/GaSe	532 nm	169	6.6 × 10^11^	[26]
MoSe_2_/Bi_2_O_2_Se	780 nm	413	3.7 × 10^10^	[27]
MoSe_2_/PdSe_2_	532 nm	651	5.29 × 10^11^	[28]

## Data Availability

Data are contained within the article and Supplementary Materials.

## Supplementary Materials

The following supporting information can be downloaded at: https://www.mdpi.com/article/10.3390/nano15120913/s1, Figure S1: Device fabrication process flow. Figure S2: Simulated in-plane electric field distributions of the metasurface under (a) 6 μm and (b) 10 μm illumination. Figure S3: *1/f* noise power spectral density (SID) of the device, *V_ds_* = 3 V. Figure S4: Device stability under 4 μm laser illumination at 3 V bias voltage.
