# Supplementary material for: Metasurface-Enhanced Infrared Photodetection Using Layered van der Waals MoSe2
Source: Nanomaterials (Basel). 2025 Jun 12;15(12):913. doi: 10.3390/nano15120913 (PMC12195908; doi:10.3390/nano15120913)
Supplement: Supplementary file 1 [file nanomaterials-15-00913-s001.zip › nanomaterials-3680576-supplementary.pdf]

Supporting Information

Metasurface-Enhanced Infrared Photodetection Using Layered van der Waals $MoSe_2$

Jinchun Li [1,2], Zhixiang Xie [2], Tianxiang Zhao [2], Hongliang Li [2], Di Wu [1,*] and Xuechao Yu [2,*]

[1] Key Laboratory of Materials Physics, Ministry of Education, School of Physics, Zhengzhou University, Zhengzhou 450001, China; jcli2023@sinano.ac.cn
[2] Key Laboratory of Multifunctional Nanomaterials and Smart Systems, Suzhou Institute of Nano-Tech and Nano-Bionics, Chinese Academy of Sciences, Suzhou 215123, China; zxxie2023@sinano.ac.cn (Z.X.); tnx.zhao@gmail.com (T.Z.); hlli2025@sinano.ac.cn (H.L.)
* Correspondence: wudi1205@zzu.edu.cn (D.W.); xcyu2022@sinano.ac.cn (X.Y.)

Photoresponsivity (R):

The calculation formula for photoresponsivity (R) is:

$$R = \frac{I_{ph}}{P_{in}}$$

where I_{ph} is the photogenerated current, P_{in} is the incident optical power.

Specific Detectivity (D*):

This parameter reflects the detector's capability to detect weak optical signals, calculated as:

$$D^* = \frac{\sqrt{A} \cdot R}{\sqrt{2e \cdot I_d}}$$

where A is the effective device area, e is the elementary charge, I_d is the dark current.

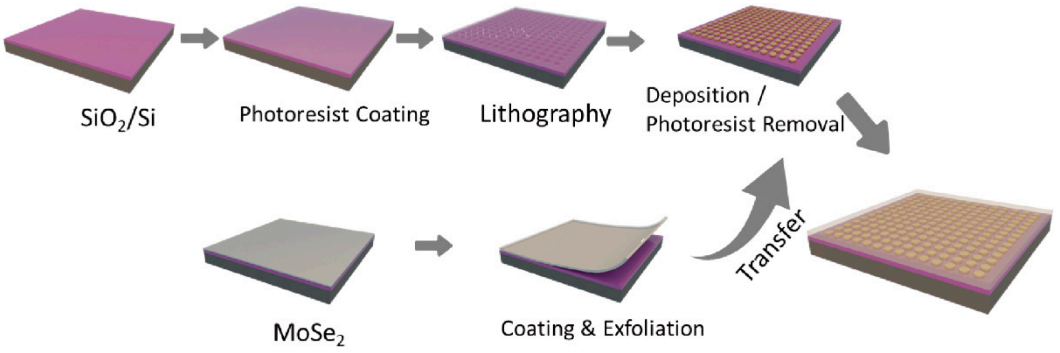

Figure S1 Device fabrication process flow.

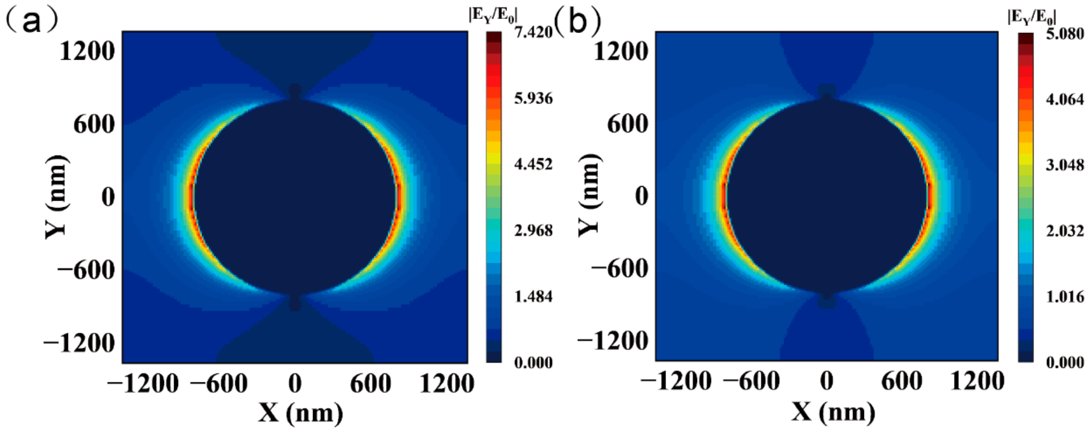

Figure S2 Simulated in-plane electric field distributions of the metasurface under (a) 6 μm and (b) 10 μm illumination.

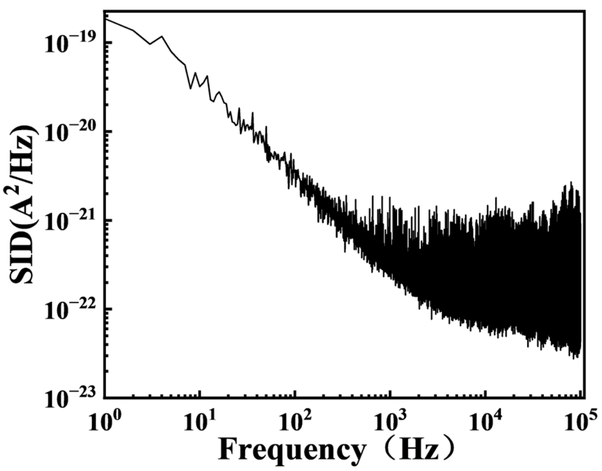

Figure S3 1/f noise power spectral density (SID) of the device, V_{ds}=3V.

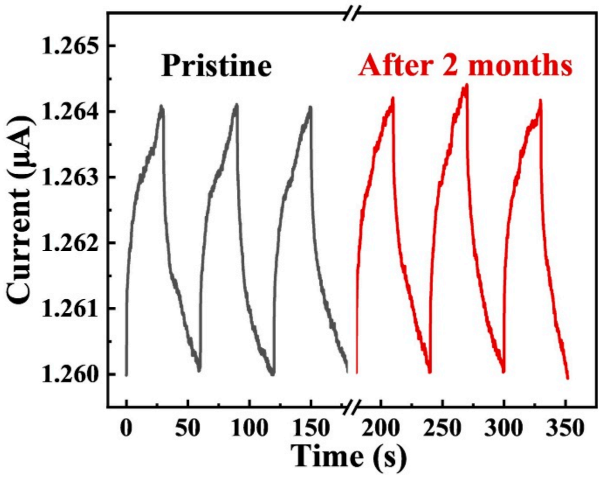

Figure S4 Device stability under 4 μm laser illumination at 3 V bias voltage.
